# Corrigendum to: MiR‐449a exerts tumor‐suppressive functions in human glioblastoma by targeting Myc‐associated zinc‐finger protein

**DOI:** 10.1002/1878-0261.13119

**Published:** 2022-01-05

Yilong *et al*. [1] contained inadvertent duplications in the cell migration and cell invasion assay images presented in Figs 3D,E and 5C,D. The authors have corrected this by providing the original images for all experimental replicates, and the revised figures and legends are included here. All authors agree to this corrigendum.

The authors apologize for any inconvenience caused.

The corrected figures are reproduced below.

**Figure:**
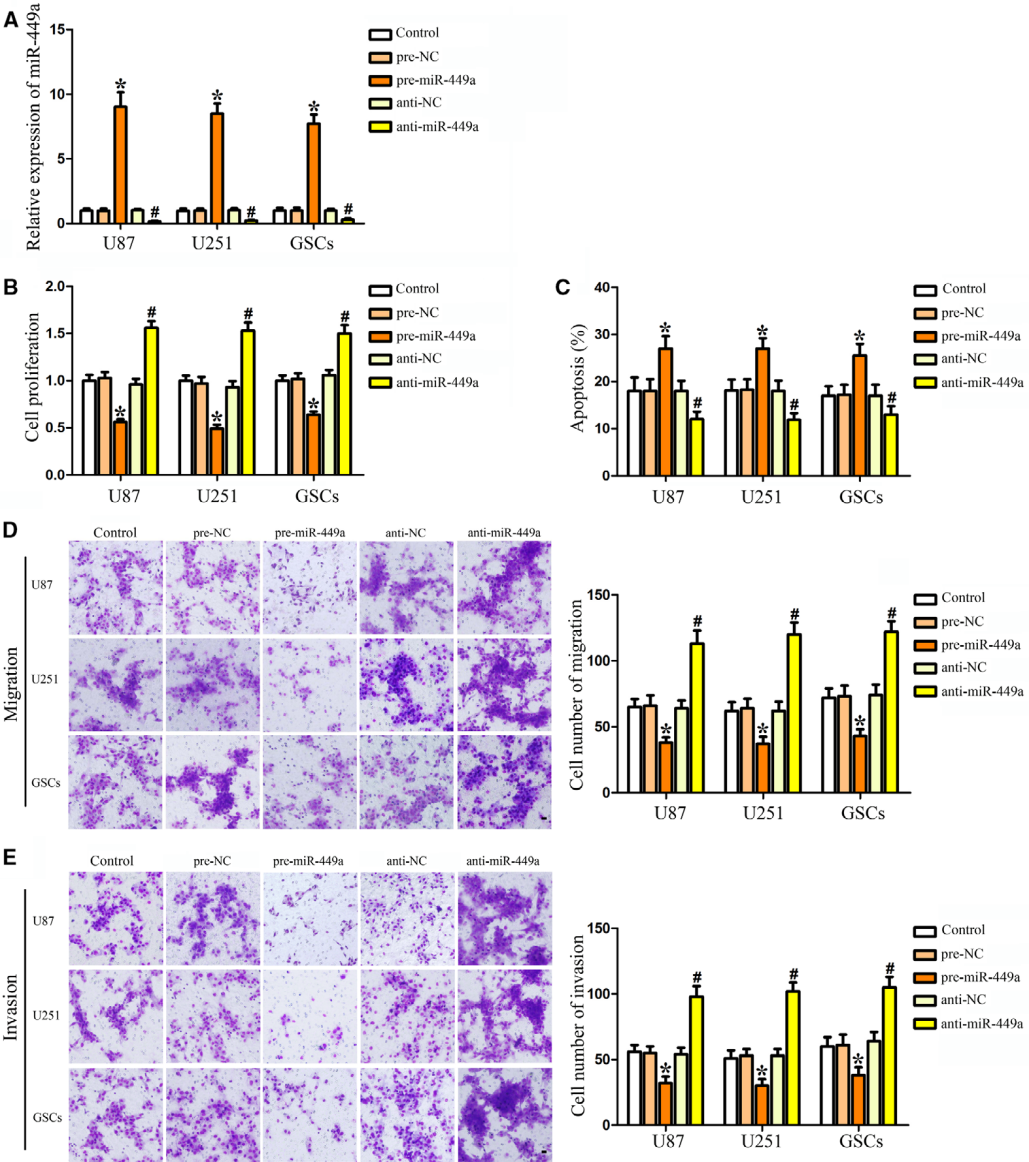
**Fig. 3**. MiR‐449a was a tumor suppressor in human GBM cell lines and GSCs. (A) Relative expression of miR‐449a after cells transfected with pre‐miR‐449a and anti‐miR‐449a. (B) Effect of miR‐449a on the proliferation of U87, U251, and GSCs. (C) Effect of miR‐449a on the apoptosis of U87, U251, and GSCs. Effect of miR‐449a on cell migration (D) and invasion (E) of U87, U251, and GSCs. Data are presented as the mean ± SD (*n* = 5, each group). *P < 0.05 vs. pre‐NC group, ^#^ *P* < 0.05 vs. anti‐NC group. Scale bars represent 20 μm.

**Figure:**
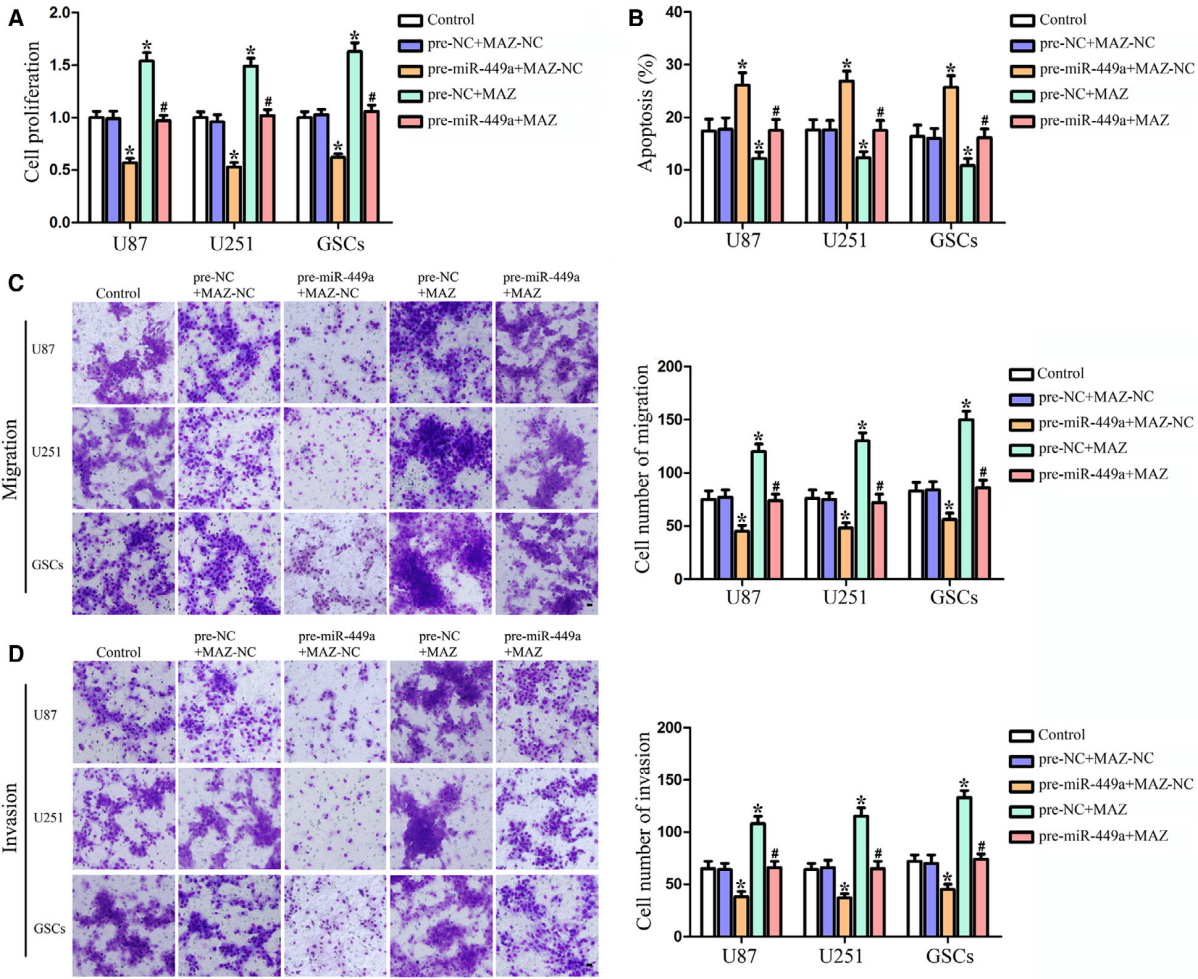
**Fig. 5.** MAZ mediated the tumor‐suppressive effects of miR‐449a on human GBM cells and GSCs. (A) CCK8 assay to evaluate the effect of miR‐449a and MAZ on the proliferation in U87, U251, and GSCs. (B) Flow cytometry analysis to evaluate the effect of miR‐449a and MAZ on the cell apoptosis in U87, U251, and GSCs. Quantification of cell migration (C) and invasion (D) to evaluate the effect of miR‐449a and MAZ on the cell migration and invasion in U87, U251, and GSCs. Representative images and accompanying statistical plots were presented. Data are presented as the mean ± SD (*n* = 5, each group). *P < 0.05 vs. pre‐NC + MAZ‐NC group, ^#^ *P* < 0.05 vs. pre‐miR‐449a + MAZ‐NC group. Scale bars represent 20 μm.

## References

[1] Yilong Y , Jun M , Yixue X , Ping W , Zhen L , Zhiqing L , Yi H , Xiuli S & Yunhui L (2015) MiR‐449a exerts tumor‐suppressive functions in human glioblastoma by targeting Myc‐associated zinc‐finger protein. Mol Oncol 9, 640–656. 25487955 10.1016/j.molonc.2014.11.003 PMC5528701

